# Adverse outcomes in COVID-19 and diabetes: a retrospective cohort study from three London teaching hospitals

**DOI:** 10.1136/bmjdrc-2020-001858

**Published:** 2021-01-06

**Authors:** Chioma Izzi-Engbeaya, Walter Distaso, Anjali Amin, Wei Yang, Oluwagbemiga Idowu, Julia S Kenkre, Ronak J Shah, Evelina Woin, Christine Shi, Nael Alavi, Hala Bedri, Niamh Brady, Sophie Blackburn, Martina Leczycka, Sanya Patel, Elizaveta Sokol, Edward Toke-Bjolgerud, Ambreen Qayum, Mariana Abdel-Malek, David C D Hope, Nick S Oliver, Vasiliki Bravis, Shivani Misra, Tricia M Tan, Neil E Hill, Victoria Salem

**Affiliations:** 1 Imperial Centre for Endocrinology, Imperial College Healthcare NHS Trust, London, UK; 2 Department of Metabolism, Digestion and Reproduction, Imperial College London, London, UK; 3 Imperial College Business School, Imperial College London, London, UK; 4 Department of Clinical Biochemistry, Imperial College Healthcare NHS Trust, London, UK; 5 Division of Medicine and Integrated Care, Imperial College Healthcare NHS Trust, London, UK

**Keywords:** type 1 diabetes mellitus, type 2 diabetes mellitus, infections, viruses

## Abstract

**Introduction:**

Patients with diabetes mellitus admitted to hospital with COVID-19 have poorer outcomes. However, the drivers of poorer outcomes are not fully elucidated. We performed detailed characterization of patients with COVID-19 to determine the clinical and biochemical factors that may be drivers of poorer outcomes.

**Research design and methods:**

This is a retrospective cohort study of 889 consecutive inpatients diagnosed with COVID-19 between March 9 and April 22, 2020 in a large London National Health Service Trust. Unbiased multivariate logistic regression analysis was performed to determine variables that were independently and significantly associated with increased risk of death and/or intensive care unit (ICU) admission within 30 days of COVID-19 diagnosis.

**Results:**

62% of patients in our cohort were of non-white ethnic background and the prevalence of diabetes was 38%. 323 (36%) patients met the primary outcome of death/admission to the ICU within 30 days of COVID-19 diagnosis. Male gender, lower platelet count, advancing age and higher Clinical Frailty Scale (CFS) score (but not diabetes) independently predicted poor outcomes on multivariate analysis. Antiplatelet medication was associated with a lower risk of death/ICU admission. Factors that were significantly and independently associated with poorer outcomes in patients with diabetes were coexisting ischemic heart disease, increasing age and lower platelet count.

**Conclusions:**

In this large study of a diverse patient population, comorbidity (ie, diabetes with ischemic heart disease; increasing CFS score in older patients) was a major determinant of poor outcomes with COVID-19. Antiplatelet medication should be evaluated in randomized clinical trials among high-risk patient groups.

**Significance of this study**

**What is already known about this subject?**

- COVID-19 is a condition caused by infection with the novel coronavirus SARS-CoV-2.
- People with diabetes are over-represented in studies of patients with COVID-19, and people with diabetes appear to have poorer outcomes in these cohorts.

**What are the new findings?**

- We demonstrate that in our large cohort of patients admitted with COVID-19 to three London teaching hospitals, diabetes is associated with increased risk of admission to intensive care unit (ICU) and/or death within 30 days of diagnosis of COVID-19 on univariate but not multivariate analysis.
- Within the entire cohort, unbiased multivariate logistic regression demonstrated that use of antiplatelet medication was associated with a reduced risk of ICU admission/death, while lower platelet count and higher Clinical Frailty Scale score were associated with an increased risk of ICU admission/death.
- Among patients with diabetes, unbiased multivariate logistic regression demonstrated that pre-existing ischemic heart disease was strongly associated with an increased risk of ICU admission/death and the protective effect of antiplatelet medication was lost.

**How might these results change the focus of research or clinical practice?**

- These results will add to the body of knowledge used to inform shielding guidance for people with diabetes, especially those with coexisting ischemic heart disease.
- Prospective randomized controlled studies of the use of antiplatelet medication in COVID-19 are required to determine if antiplatelet medication improves outcomes in patients with COVID-19 and if this remains true for patients with established vascular complications of the metabolic syndrome.

## Introduction

In February 2020, the first cohort studies from Wuhan reported clinical outcomes of patients treated for COVID-19.^1–5^ Patients with hypertension and ischemic heart disease (IHD) were over-represented in hospital or intensive care unit (ICU) admissions,^1 3 4^ and some studies have reported that patients with these comorbidities are more likely to have poorer outcomes even after adjustment for age and smoking status.^6^

The prevalence of diabetes in patients admitted to ICU for severe COVID-19 (typically 20%–30%)^3 5^ greatly exceeds the adult population prevalence of diabetes (ie, 6.8% in the UK).^7^ Patients infected with the prior two human coronaviruses (severe acute respiratory syndrome coronavirus and Middle East respiratory syndrome coronavirus) were more likely to experience more severe disease and die if they had a diagnosis of diabetes.^8 9^ However, granularity on the way diabetes interacts with the natural history of COVID-19 has been slower to come and remains an area of intense concern for patients with diabetes.^10^

Two major hospital cohort studies provided early insight about the interaction between diabetes and COVID-19. Zhu *et al*^11^ concluded that good glycemic control in the acute hospital setting in China was an important factor for better outcomes in patients with pre-existing type 2 diabetes, although it was difficult to ascertain whether this was confounded by the possibility that poorer glycemia in hospital was a marker of a more severe inflammatory response or the decision to use corticosteroids. The Coronavirus SARS-CoV-2 and Diabetes Outcomes (CORONADO) study reported that on multivariate analysis of patients with diabetes hospitalized for COVID-19, body mass index (BMI), but not glycated hemoglobin (HbA1c), was positively and independently associated with poorer outcomes.^12^ More recently, a population-based study from England indicated that higher HbA1c increased the risk of mortality of patients with COVID-19 and diabetes, while the relationship between BMI and mortality was U-shaped.^13^

Here we provide a report of an unbiased/unselected multivariate analysis of all patients hospitalized with swab-positive COVID-19 in three London teaching hospitals, under the umbrella of Imperial College Healthcare NHS Trust (ICHNT), during the initial peak period of infections.

## Research design and methods

### Study setting

We performed this retrospective cohort study at ICHNT, which includes three hospitals admitting patients with COVID-19 (Charing Cross Hospital, Hammersmith Hospital and St Mary’s Hospital). All patients who had a nasopharyngeal swab taken to determine SARS-CoV-2 infection between March 9 and April 22, 2020 were identified on an automated search of the laboratories’ computer systems. COVID-19 diagnosis was determined by the presence of SARS-CoV-2 infection as evidenced by a positive reverse transcription-PCR (RT-PCR) result from a nasopharyngeal swab. RT-PCR was performed on nasopharyngeal swabs by North West London Pathology staff in the laboratories in the constituent hospitals of ICHNT.

**Figure 1:**
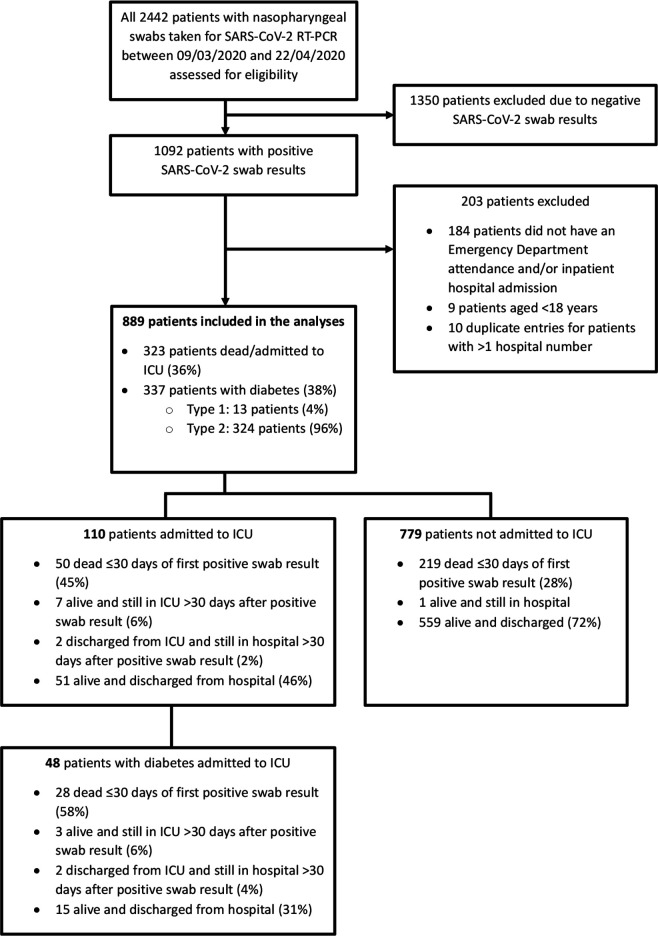
All 2442 patients who had nasopharyngeal swabs taken for RT-PCR to detect the presence of SARS-CoV-2 (and diagnose COVID-19) between March 9 and April 22, 2020 were assessed for eligibility. 1350 patients with negative swab results were excluded. Of 1092 patients with positive swab results, 203 were excluded (as they did not have an emergency department attendance and/or inpatient hospital admission or they were aged less than 18 years), and 889 patients were included in the statistical analyses. ICU, intensive care unit; RT-PCR, reverse transcription-PCR.

Excluded from data collection were all patients with negative SARS-CoV-2 swab results, patients who did not have an emergency department attendance and/or an inpatient admission within ICHNT, and all patients aged less than 18 years (figure 1). For patients with more than one positive swab result or multiple hospital admissions during the data collection period, the date of the earliest swab result and the most significant hospital admission in terms of COVID-19 severity, respectively, were used for data collection.

### Data collection

Hospital numbers of patients with positive swab results were checked against electronic health records held on the ICHNT computer system (Cerner Corporation, Kansas City, USA). Data are uploaded onto Cerner manually by health practitioners and administrators to record and store patient notes, prescribe and dispense medication, and store investigation results and clinic letters. Furthermore, Cerner is connected to the National Health Service (NHS) Spine, which synchronizes other data (such as date of death).

Demographic, clinical and biochemical data (on the date of admission/emergency department attendance or on the date of the first positive SARS-CoV-2 swab result if the patient had been admitted for ≥1 week), as well as outcome data, were collected for all eligible patients up to and including June 20, 2020 to ensure there was a minimum of 30 days of follow-up after the date of COVID-19 diagnosis to eliminate the issue of censoring.

Data collected included demographic and clinical characteristics (age, gender, ethnicity, most recent body weight), specific comorbidities (including classification and duration of diabetes) and medications on admission. A score using the Clinical Frailty Scale (CFS)^14^ was assigned to every patient. Hematological and biochemical data on the day of COVID-19 diagnosis included full blood count, biochemistry and arterial blood gas results. Average capillary blood glucose (CBG) values were recorded for the first 3 days following COVID-19 diagnosis and the most recent HbA1c result (within 6 months) was recorded.

### Statistical analysis

The composite primary outcome was admission to ICU or death within 30 days of COVID-19 diagnosis, and data on the primary outcome were collected for all patients. This was chosen to capture patients with severe and/or life-threatening COVID-19. Univariate and then unrestricted multivariate logistic regression analyses were performed to look for factors associated with an increased risk of the primary outcome. Unlike a Cox proportional hazards approach, this analysis focuses more on ‘if’ a patient gets severely ill rather than survival duration.

The first stage of analysis was descriptive. Quantitative data are expressed as mean±SD. Categorical variables were given as percentage (number) of participants. Univariate logistic regression models were used to calculate OR associated with the primary outcome. Comparison between two groups was analyzed using Student’s t-tests (normally distributed data) or Mann-Whitney U test (non-normally distributed data) for continuous variables. Comparison of categorical variables was analyzed by Fisher’s exact test or χ^2^ test. A difference with a two-sided α<0.05 was considered statistically significant.

Multivariable logistic regression was applied to assess the independent association of the primary outcome with all the clinical and biological features included in the study. For the top level (unrestricted) analysis only data sets with <5% missing values were included, leaving a total number of 716 patients and 37 predictors (variables). No data points were imputed. Data were winsorized (at 1% level) to neutralize the effects of the most extreme values. Following this first step, a regularized regression (smoothly clipped absolute deviation, SCAD) analysis^15–17^ was performed to independently account for the overdimensionality of the unrestricted multivariate. This sensitivity analysis allows unbiased selection of the most relevant and important predictors and controlling the errors involved in variable selection, without the need to re-estimate the model in a stepwise fashion. Results are presented as the estimate parameter, the p value of the association and the marginal effect size.

Next, only patients with diabetes were included in multivariate and regularized regression analyses to look for elements of their clinical presentation that were associated with poorer outcome with COVID-19. Finally, marginal effects for the most significant (independent) risk factors were substratified by age (for the CFS) and the presence or absence of IHD or usage of antiplatelet agents (for platelet counts). All statistical analyses were performed on GraphPad Prism V.8.1 (descriptive statistics) and custom-written MaTLAB scripts (multivariate analyses). A detailed description of statistical methods can be found in the online supplemental material.

10.1136/bmjdrc-2020-001858.supp1 Supplementary data

## Results

### Study cohort

We report on 889 consecutive adult patients with a confirmed (SARS-CoV-2 swab-positive) diagnosis of COVID-19 admitted to three central London hospitals within the same NHS Trust (ICHNT) between March 9 and April 22, 2020. A total of 323 (36%) of these patients met the primary outcome of death or admission to the ICU within 30 days of diagnosis. In the cohort of patients studied here, 337 of 889 (38%) had a diagnosis of diabetes, 96% (324 of 337) of people with diabetes had type 2 diabetes (including 4 patients who were diagnosed during their admission for COVID-19) and the remainder (13 patients) had type 1 diabetes. The study flow diagram is shown in figure 1.

### Factors prior to hospital admission

The characteristics of the entire cohort are shown in online supplementary table S1. Of all patients treated for COVID-19 in our NHS Trust, the average (±SD) age was 65.8 (±17.5) years and 60% were men. The average (±SD) weight for men was 80.8 (±19.8) kg and for women 72.6 (±24.2) kg. Of our cohort, 62% were from non-white ethnic background, including 17% black and 11% South Asian. The most common comorbidities were hypertension (47%), hypercholesterolemia (43%), diabetes (38%) and IHD (16%).

By comparison, among the patients who had diabetes treated for COVID-19 (online supplementary table S2), the average age was 68.5±14.6 years and 66% were men. The average weight was 83.5 (±18.9) kg for men and 77.8 (±24.5) kg for women. In this cohort of patients with diabetes, 72% were non-white, including 20% black and 14% South Asian. Other metabolic comorbidities were much more common in patients with diabetes compared with the whole cohort: a medical history of hypertension was present in 70%, IHD in 28% and hyperlipidemia in 51%. The average HbA1c was 64.7 (±21.7) mmol/mol (8%), 27% were taking an ACE inhibitor and 63% were taking a statin.

### Factors at the time of diagnosis of COVID-19

Online supplemental table 3 summarizes the differences in presenting clinical characteristics between patients who fulfilled the primary outcome compared with patients who survived and were not admitted to ICU. Online supplemental table S4 compares the presentation features between patients with a diagnosis of diabetes and those without diabetes. Online supplemental table S5 shows the difference in presentation characteristics for patients with diabetes stratified by the primary outcome compared with patients with diabetes who survived and were not admitted to ICU. Biochemical parameters established to be associated with adverse outcomes, including procalcitonin and D-dimers, were significantly higher in the group who fulfilled the primary outcome of death/ICU admission (online supplemental table S3). The mean values for procalcitonin were slightly lower in patients with diabetes compared with those without diabetes (online supplemental table S4). However, among patients with diabetes, higher procalcitonin levels were still a predictor of poorer outcomes (online supplemental table S5). For both patients with diabetes and those without, a CBG below 10 mmol/L at presentation was associated with a lower risk of poor outcome. In patients without diabetes, this association was sustained after correction for age and gender; however, this was not sustained on multivariate analysis in patients with diabetes (see table 1).

**Table 1:** Unbiased multivariate logistic regression analysis of 42 regressors (variables) against the primary outcome of death/ICU admission within 30 days of COVID-19 diagnosis in patients with diabetes mellitus (n=268 patients)

Regressor	Estimate	SE	P value	Marginal effect (%)
Age	0.059	0.019	**0.001**	**1.3**
Male gender	0.827	0.453	0.068	17.9
Ethnicity: South Asian	−1.352	0.736	0.066	−29.2
Ethnicity: black	0.216	0.578	0.709	4.7
Ethnicity: white	−0.091	0.508	0.858	−2.0
Type 1 diabetes	0.993	1.135	0.382	21.5
Active foot disease	0.075	0.839	0.929	1.6
Stroke	0.338	0.523	0.519	7.3
Hyperlipidemia	−0.503	0.447	0.261	−10.9
Ischemic heart disease	1.547	0.569	**0.007**	**33.4**
Heart failure	−0.956	0.613	0.119	−20.7
Hypertension	−0.101	0.504	0.841	−2.2
Chronic obstructive pulmonary disease	−0.499	0.661	0.450	−10.8
Active cancer	−0.383	0.727	0.598	−8.3
Insulin	0.228	0.992	0.818	4.9
GLP-1 receptor agonist	1.040	1.755	0.553	22.5
Metformin	−0.064	0.918	0.944	−1.4
Sulfonylurea	0.475	0.991	0.632	10.3
Dipeptidyl peptidase-IV inhibitor	−0.825	0.935	0.378	−17.8
Total number of medications for diabetes	0.137	0.800	0.864	3.0
Statin	−0.105	0.504	0.835	−2.3
ACE inhibitor	0.536	0.513	0.297	11.6
Angiotensin II receptor blocker	0.200	0.529	0.705	4.3
Antiplatelet drug	−0.735	0.486	0.130	−15.9
White cell count	−0.112	0.146	0.442	−2.4
Hemoglobin	−0.018	0.012	0.130	−0.4
Platelet count	−0.009	0.003	**0.001**	−**0.2**
Neutrophils	0.123	0.140	0.376	2.7
Lymphocytes	0.093	0.406	0.819	2.0
Serum sodium	0.077	0.039	**0.050**	**1.7**
eGFR on diagnosis	−0.012	0.010	0.224	−0.3
C reactive protein	0.002	0.002	0.536	0.0
Capillary blood glucose on diagnosis	0.034	0.038	0.369	0.7
Temperature	0.017	0.206	0.935	0.4
Respiratory rate on diagnosis	0.047	0.041	0.252	1.0
Heart rate on diagnosis	−0.004	0.014	0.774	−0.1
Systolic blood pressure	0.003	0.010	0.793	0.1
Diastolic blood pressure	0.015	0.017	0.391	0.3
NEWS on diagnosis	−0.001	0.110	0.990	0.0
Inspired oxygen delivered on diagnosis	0.000	0.011	0.974	0.0
Oxygen saturations on diagnosis	0.020	0.036	0.575	0.4
Maximum inspired oxygen required	0.072	0.012	**<0.0001**	**1.6**

This is an unselected multivariate logistic (logit) analysis of all variables that were collected for patients admitted with swab-positive COVID-19 who had diabetes mellitus, as applied to the primary outcome of death or ICU admission within 30 days. 268 patients are included with 42 variables, with the only exclusions being those patients/variables for which ≥5% data points were unknown. For this reason, HbA1c is not included as a regressor as it would have reduced the number of patients included to 168, although of note in that regression HbA1c did not survive multiple correction (and neither did body weight). For categorical variables, a positive ‘estimate’ indicates an increased risk of the primary outcome (death or ICU admission) with that variable present, and a negative estimate indicates a reduced risk of the primary outcome if that variable is present. The p value is a measure of the confidence of that variable being an independent predictor of the primary outcome corrected for all of the other regressors listed. For continuous variables, a positive ‘estimate’ indicates an increasing risk of the primary outcome as the variable increases. Since in logistic regressions estimated coefﬁcients cannot be interpreted as a measure of the contribution of the effect, we have also calculated marginal effects along with their SEs. A positive marginal effect indicates that an increase in that variable is associated with a fully adjusted increased risk of the primary outcome. The converse applies for negative marginal effects. For categorical variables, the marginal effect indicates the percentage increased risk of the primary outcome, if that variable exists. So, for example, patients with diabetes (all other things being equal) have a 33% increased risk of death/ICU if they have IHD.

Statistically significant P values are shown in bold.

eGFR, estimated glomerular filtration rate; GLP-1, glucagon-like peptide-1; HbA1c, glycated hemoglobin; ICU, intensive care unit; IHD, ischemic heart disease; NEWS, National Early Warning Score.

Compared with patients who did not have diabetes, patients with diabetes had significantly lower arterial blood gas pH values, lower bicarbonate levels and higher serum potassium levels (although still, on average, within the laboratory reference range) at the time of COVID-19 diagnosis (online supplemental table S4). This hints at the possibility that patients with diabetes were more likely to have diabetic metabolic emergencies at presentation. Indeed, in our cohort there were 59 episodes of diabetic ketoacidosis associated with COVID-19 during the data collection period. However, admission measurements of acidemia were not significantly lower in patients with diabetes who met the primary outcome compared with patients who survived and were not admitted to ICU (online supplemental table S5).

Chest radiographs on diagnosis were reported by inhouse radiologists on a 6-point severity scale (0=normal, 5=widespread, dense bilateral infiltrates). Of the patients who survived without ICU admission, 60% had the classic patchy ground glass changes, with a median severity score of 2 out of 5. Among those who died/were admitted to ICU, 72% had the classic radiological features, with a median severity score of 3. These proportions were similar among the patients with diabetes. The frequency distribution of chest X-ray severity score by primary outcome, with poorer outcomes associated with higher chest X-ray severity scores, was highly significantly different on χ^2^ testing with 5 df (p<0.0001).

### Clinical features of the whole cohort associated with the primary outcome (death or ICU admission within 30 days of COVID-19 diagnosis)

On univariate analysis, the risk of the primary outcome was significantly higher in men and older age groups (online supplemental table S1). Hypertension, IHD, pre-existing renal impairment and heart failure were each associated with an increased risk of poor outcome on univariate analysis. Patients taking an ACE inhibitor had an OR of 1.57 (CI 1.10 to 2.22, p=0.013) for the primary outcome, but an increased risk was not statistically evident with patients on angiotensin receptor blocker drugs. Furthermore, the CFS score (ranging from 1 (very fit) to 9 (terminally ill)) was a good predictor of poorer outcomes, with patients scoring ≥7 having an OR of 2.34 (1.46 to 3.76, p=0.0004) for death/ICU admission compared with those scoring 1–2. Weight >90 kg did not reach significance for predicting poorer outcomes referenced against patients who weighed between 60 kg and 90 kg. However, patients weighing less than 60 kg were more likely to have poorer outcomes (online supplemental table S1).

After removing data sets where >5% data points were missing (ie, 170 patients), we ran an unselected multivariate logistic regression analysis of 37 regressors (variables) against the primary outcome on 719 patients (table 2). Age and gender retained significance as independent variables. As well as reporting p values for the significance of the independent contribution of a given variable, we have also calculated marginal effect sizes. This percentage value is a measure of the altered risk of primary outcome (death/ICU admission) as the given variable changes. The marginal effect of male gender was +9.3%; that is, all other factors being the same, the risk of primary outcome was 9.3% higher for men than for women. The CFS score was also found to be a significant independent predictor of death/ICU admission. Each unit rise in score (from 1 to 9) was associated with a 3.1% increased risk of death/ICU admission, all other things being equal.

**Table 2:** Unbiased multivariate logistic regression analysis of 37 regressors (variables) against the primary outcome of death/ICU admission within 30 days of COVID-19 diagnosis (n=719 patients)

Regressor	Estimate	SE	P value	Marginal effect (%)
Age	0.029	0.011	**0.005**	**0.5**
Male gender	0.553	0.256	**0.031**	**9.3**
Ethnicity: South Asian	−0.321	0.441	0.466	−5.4
Ethnicity: black	−0.296	0.360	0.411	−5.0
Ethnicity: white	−0.179	0.272	0.510	−3.0
Diabetes mellitus	0.099	0.265	0.709	1.7
Stroke	−0.457	0.348	0.189	−7.7
Hyperlipidemia	0.117	0.277	0.673	2.0
Ischemic heart disease	0.596	0.364	0.102	10.1
Heart failure	0.046	0.408	0.910	0.8
Hypertension	0.207	0.285	0.468	3.5
Chronic obstructive pulmonary disease	−0.051	0.384	0.893	−0.9
Active cancer	−0.135	0.428	0.752	−2.3
Statin	−0.310	0.288	0.282	−5.2
ACE inhibitor	0.351	0.329	0.285	5.9
Angiotensin II receptor blocker	0.145	0.366	0.691	2.5
Antiplatelet drug	−0.616	0.319	**0.053**	−**10.4**
Clinical Frailty Scale score	0.183	0.078	**0.019**	**3.1**
White cell count	0.031	0.085	0.720	0.5
Hemoglobin	−0.008	0.006	0.182	−0.1
Platelet count	−0.004	0.001	**0.001**	−**0.1**
Neutrophils	0.009	0.086	0.920	0.1
Lymphocytes	−0.011	0.229	0.963	−0.2
Serum sodium	0.072	0.023	0.002*	**1.2**
Serum potassium	0.202	0.194	0.300	3.4
eGFR on diagnosis	−0.006	0.005	0.287	−0.1
C reactive protein	0.002	0.001	0.132	0.0
Temperature	−0.012	0.113	0.913	−0.2
Respiratory rate	0.034	0.021	0.103	0.6
Heart rate	0.006	0.008	0.453	0.1
Systolic blood pressure	0.010	0.006	0.088	0.2
Diastolic blood pressure	−0.002	0.010	0.831	0.0
National Early Warning Score	−0.015	0.055	0.790	−0.2
Inspired oxygen delivered on diagnosis	−0.012	0.006	**0.046**	−**0.2**
Oxygen saturations on diagnosis	−0.032	0.018	0.079	−0.5
Maximum inspired oxygen required during admission	0.068	0.007	**<0.0001**	**1.2**

This is an unselected multivariate logistic (logit) analysis of all variables that were collected for patients admitted with swab-positive COVID-19. 719 patients are included with 37 variables, with the only exclusions being those patients/variables for which ≥5% data points were unknown. For categorical variables, a positive ‘estimate’ indicates an increased risk of the primary outcome (death or ICU admission) with that variable present, and a negative estimate indicates a reduced risk of the primary outcome if that variable is present. The p value is a measure of the confidence of that given variable being an independent predictor of the primary outcome corrected for all of the other regressors listed. For continuous variables, a positive ‘estimate’ indicates an increasing risk of the primary outcome as the variable increases. Since in logistic regressions estimated coefﬁcients cannot be interpreted as a measure of the contribution of the effect, we have also calculated marginal effects. A positive marginal effect indicates that an increase in that variable is associated with a fully adjusted increased risk of the primary outcome. The converse applies for negative marginal effects. For categorical variables, the marginal effect indicates the percentage increased risk of the primary outcome, if that variable exists. So, for example, all other variables being equal, men have a 9.3% increased risk of death or ICU admission than women.

Statistically significant P values are shown in bold.

*The effect of serum sodium was skewed by patients with serum sodium >145 mmol/L.

eGFR, estimated glomerular filtration rate; ICU, intensive care unit.

The association between ethnicity and poor outcome was not sustained on multivariate analysis in our cohort. None of the individual comorbidities we collected data on (ie, diabetes, hypertension, hyperlipidemia, IHD, cerebrovascular disease, heart failure, chronic obstructive airway disease and cancer) were found to be independent predictors of mortality/ICU admission in our cohort, including diabetes.

Taking an antiplatelet drug was significantly and independently associated with a 10% lower risk of death and/or ICU admission (table 2). Related to this, platelet count was a significant predictor of poor outcome. Our data suggest that every reduction in platelet count of 100×10^9^/L is associated with a 10% risk of death/ICU admission. This association remained after removal of patients with platelet counts below 50×10^9^/L (the majority of whom had active, often hematological, malignancies). On univariate analysis, patients taking antiplatelet agents did not have different outcomes from those who were not taking antiplatelet agents (online supplemental table S1), although this is likely due to the fact that any beneficial effects of the drug are masked by their particular usage in high-risk groups, for example, those with IHD. Among patients with a pre-existing diagnosis of IHD, 51% of those who were *not* taking an antiplatelet drug died or were admitted to the ICU, compared with 53% of those with IHD who were taking an antiplatelet agent. Furthermore, in patients with diabetes, taking an antiplatelet agent was associated with a negative parameter estimate that was not statistically significant (table 1). Taken together, this suggests that antiplatelet agents may not protect as well against the platelet-consuming coagulopathy of severe disease in particularly high-risk groups.

Higher serum sodium at the time of COVID-19 diagnosis was also found to be a strong independent predictor of poor outcome; however, this association disappeared after removing patients with high sodium (ie, >145 mmol/L). As a sensitivity analysis, we performed a regularized regression (SCAD), which selects the truly significant variables in the multivariate regression without the limitations of a stepwise approach (see online supplemental material). This SCAD analysis identified 23 variables driving the variance (online supplemental table S6). It supported the findings of the unrestricted multivariate regression (table 2).

### Clinical features of patients with diabetes associated with the primary outcome

Unrestricted multivariate logistic analysis with >95% complete data sets was possible on 278 patients with diabetes (table 1). This excluded HbA1c from the analysis due to inadequate data. However, multivariate analysis on 197 patients for HbA1c did not produce a significant interaction with the primary outcome (p>0.9) and neither was it a significant factor on univariate analysis (online supplemental table S2). Of all the associated comorbidities examined, IHD had a highly significant 33% marginal effect for increased likelihood of death/ICU admission for patients with diabetes. IHD was present as a comorbidity in 28% of patients with diabetes compared with 16% of the whole cohort. On univariate analysis, IHD was associated with a relative risk of 1.2 (p=0.02) for poor outcome in patients with diabetes compared with patients with diabetes who did not have IHD (online supplemental table S2). Conversely, in all patients with IHD, outcomes were already poor (51% of patients with pre-existing IHD died or were admitted to ICU), and this was not further impacted on by the additional diagnosis of diabetes. Taken together this suggests that it is not diabetes per se, but its association with other cardiometabolic conditions (particularly established IHD) that confers risk of poorer outcomes with COVID-19. Other factors significantly and independently associated with poor outcome in patients with diabetes were age, lower platelet count and maximum inspired oxygen required during admission (table 1). In patients with diabetes, male gender was no longer a significant independent predictor of poorer outcome, although there was a trend for this to remain the case (p=0.07). This was corroborated on SCAD analysis (online supplemental table S7).

### Stratified marginal effect size for the significant predictors that survived multiple logistic regression: platelet count and CFS score

**Figure 2:**
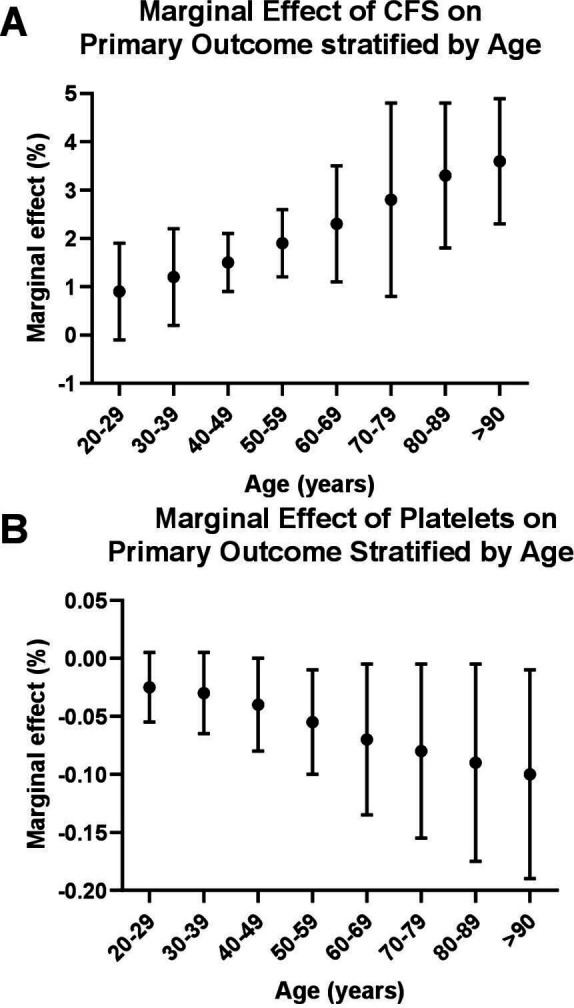
On unbiased univariate analysis of 798 patients admitted with COVID-19, the Clinical Frailty Scale (CFS) score was a significant independent predictor of poor outcomes, with a marginal effect size of 3% for every increase in score between 1 and 9 (see table 2). We stratified the marginal effect by age bands. There was a trend (A) for the marginal effect of the CFS score on the primary outcome to be greater in the older population. Similarly, there was a trend for the independent marginal effect of platelet count on the primary outcome to increase with age (B), but there was no interaction with the marginal effect size of platelet count on outcomes as stratified by the use of antiplatelet drugs (data not shown).

We stratified the marginal effect of CFS score as an independent predictor of poor outcome in COVID-19 by age group (figure 2A). This confirms that CFS score is a useful predictor of the primary outcome in older age groups. The independent marginal effect of platelet count tends to increase with age (figure 2B), but there was no interaction with the use of antiplatelet drugs (p>0.9).

## Discussion and conclusions

Here we report on a large cohort of patients diagnosed with COVID-19 over a 6-week period in a multisite NHS Trust in London. In our cohort, multivariate analysis revealed male gender, increased age, increased frailty and lower platelet count were independently associated with increased risk of ICU admission and/or death within 30 days of COVID-19 diagnosis, while taking antiplatelet medication was associated with a lower risk of poor outcome. Within the subset of our cohort with diabetes (96% type 2 and 4% type 1), multivariate analysis demonstrated pre-existing IHD, advancing age and lower platelet count were associated with increased risk of ICU admission and/or death.

Prior to the COVID-19 pandemic, data from the National Diabetes Inpatient Audit indicate the prevalence of diabetes among hospital inpatients in England and Wales was 18%,^18^ reflecting a generalized increased risk of hospitalization among people with diabetes. Using data from a UK primary care database, Barron *et al*^19^ reported that people with both type 1 and type 2 diabetes had multiply adjusted increased odds of dying in hospital with COVID-19 compared with those without diabetes. Our results show that people with diabetes are at increased risk of severe or life-threatening COVID-19, although this was driven by its tendency to coexist with other conditions, particularly IHD. The relative proportions of patients admitted with COVID-19 with type 1 and type 2 diabetes were similar to the population we serve, suggesting no difference in susceptibility based on diabetes type.

Hyperglycemia is a modifiable factor that may influence outcome in COVID-19, especially in people with diabetes. In our cohort, recent HbA1c was not a significant predictor of poor outcome, which is similar to some cohort studies^12^ but not others.^13^ In patients with diabetes (and in patients without diabetes), our data demonstrate that blood glucose ≥10 mmol/L (at the time of COVID-19 diagnosis and on average during the 72 hours following COVID-19 diagnosis) is associated with increased risk of death/ICU admission. Similar findings have been reported by groups from England,^13^ France^12^ and China.^11 20^ This association is not sustained on multivariate analysis in our patients with diabetes and there is no prospective study to address whether maintaining blood glucose <10 mmol/L would improve outcomes for patients with diabetes with COVID-19. Furthermore, maintaining blood glucose <10 mmol/L may become even more challenging to achieve as the use of dexamethasone in the management of severe COVID-19 becomes more widespread, following publication of beneficial reports of its use in this context.^21^

It is now well established that patients with more severe manifestations of COVID-19 (including those that need to be escalated to ICU care and those that die) are much more likely to have a diagnosis of diabetes than those who are documented as having a mild form of the infection.^2 19 22^ Some people have recommended that patients with diabetes need to be more actively shielded, and diabetes may be associated with a higher risk of viral infection.^23^ Evidence of increased risk of contracting COVID-19 in people with diabetes is lacking, with similar adult prevalence of diabetes (10.1%) and prevalence of diabetes in patients with COVID-19 (10.9%) reported by the Centers for Disease Control in America.^24^ However, it is important to note that here we show that diabetes alone is not a major factor contributing to the risk of death/ICU admission, but rather its association with other consequences of the metabolic syndrome, particularly IHD, confers a higher risk of a poor outcome. For instance, patients (in our cohort) with diabetes have a 33% increased risk of death/ICU admission if they also have IHD.

In line with our observations that it is multimorbidity per se and not any single particular diagnosis that confers a strong increased risk of poorer outcomes in COVID-19 infection, we show that the CFS score is a robust, independent predictor of poor outcome on multivariate analysis. This is consistent with (COVID-19 and non-COVID-19) studies that show that as the CFS score increases, the likelihood of mortality increases.^25 26^ However, we have also shown that the CFS score is much less useful in younger age groups. We chose death or ICU admission as our primary outcome measure as this incorporated all patients with severe/life-threatening COVID-19. Our cohort also included those who, with unfavorable chances of tolerating and surviving invasive ventilation, would not have been admitted to ICU due to pre-existing multimorbidity.

We report a strongly significant and independent risk of death/ICU admission as platelet count at presentation decreases. The inverse relationship between platelet count and risk of death/ICU admission with COVID-19 has also been reported by several other studies.^22 27 28^ As microvascular and macrovascular thrombosis is increasingly being reported as a feature of severe COVID-19,^29–31^ reduced platelet count may reflect consumptive coagulopathy (D-dimers were significantly higher in the primary outcome group), possibly in conjunction with direct effects of the virus on thrombopoiesis or platelet survival. We note that patients with coexisting diabetes and IHD are at particular risk of poorer outcomes with COVID-19, and that in these patients the protective benefits of antiplatelet usage are abrogated. Differing recommendations about the use of anticoagulants in hospitalized patients with COVID-19 have been made in recently published international guidelines,^32^ while several prospective randomized trials evaluating the effects of anticoagulation on COVID-19 mortality are currently underway.

Strengths of our study include a diverse population of patients and indepth characterization, including CFS and numerous prehospital and presentation factors. We selected a statistical approach focused on a primary outcome measure with no selection bias for the multivariate analysis that produced intuitive results, which survived robust sensitivity analysis.

Large population studies (of millions of patients), using primary care databases in England, have reported increased mortality risk among patients with COVID-19 who have diabetes.^19 33^ Similarly, several meta-analyses (of thousands of patients) have reported increased risk of poor outcomes and/or death in people with COVID-19 who have diabetes.^34 35^ In our cohort, diabetes per se was not associated with an increased risk of ICU admission and/or death on multivariate analysis (which included 37 variables including presenting clinical features as well as pre-existing comorbidities). It is clear that patients with diabetes are over-represented among those that have the poorest outcomes with COVID-19, and our findings suggest that this is extensively driven by the association of diabetes with cardiovascular disease.

In conclusion, this study contributes further to understanding the drivers of poor outcomes in patients with diabetes admitted to hospital with COVID-19. Advancing age, multimorbidity (as crystallized in the CFS) and lower platelet count are important predictors of poor outcome in patients with COVID-19 admitted to hospital. The protective benefits of usage of antiplatelet agents are lost in patients with diabetes. There is no clear evidence that dysglycemia drives the increased risk of death/ICU admission among patients with diabetes, but rather the association of diabetes with other common medical conditions confers excess risk of poor outcomes.
