# Supplementary material for: Adverse outcomes in COVID-19 and diabetes: a retrospective cohort study from three London teaching hospitals
Source: BMJ Open Diabetes Res Care. 2021 Jan 6;9(1):e001858. doi: 10.1136/bmjdrc-2020-001858 (PMC7789097; doi:10.1136/bmjdrc-2020-001858)
Supplement: Supplementary data [file bmjdrc-2020-001858supp001.pdf]

BMJ Publishing Group Limited (BMJ) disclaims all liability and responsibility arising from any reliance placed on this supplemental material which has been supplied by the author(s)

<u>Supplementary Methods</u>

STATISTICAL ANALYSES

Multiple logistic regression was chosen as the natural approach to model the probability of a binary outcome (death or ICU admission within 30 days of diagnosis). To neutralise the effects of extreme values all continuous variables were winsorized, at the 99% level in the case of the regression on all patients and at 97% in the case of the cohort of patients with diabetes (given their reduced sample size). We ran the first regression including all the potentially relevant predictors to avoid the problem of omitted variable bias. Estimated coefficients can be inconsistent if a relevant regressor is selected out of the model. Unlike in linear models, estimated coefficients in logistic regressions cannot be interpreted as marginal effects. The latter can be calculated alongside their standard errors. For example, the marginal effect of a generic regressor Xi measures the expected change on Pr (primary outcome measure) after a unit increase of Xi, holding all other Xs at their sample averages.

To enhance the interpretability and select the truly significant variables in our regression model, we have resorted to a regularised regression, which solves a general problem of the type:

$$\min_{\beta_0,\beta}\left(\frac{1}{2n}\sum_{i=1}^{n}\left(y_i-\beta_0-x_i^\top\beta\right)^2+\sum_{j=1}^{p}p_\lambda\left(|\beta_j|\right)\right)$$

where *yi* is the response variable, *xi* is the vector of *p* predictors, *n* is the number of observations and the *β*s are the parameters to estimate. The main difference between Lasso and the standard minimization involved in least squares regressions is the presence of the “penalty” λPp j=1|βj|, where λ is a non-negative tuning parameter. This penalty reduces the number of regressors and, therefore, serves the purpose of regularizing the problem and selecting the important predictors at the same time.

One of the first examples of regularized regression techniques is LASSO, popularized in the statistical literature by Tibshirani [1] and used in medical statistics.[2, 3] In many empirically relevant scenarios, the LASSO has been shown to perform better than stepwise selection methods.[4] In this paper we have resorted

to the SCAD penalty proposed by Fan et al.,[5] which has two important advantages: 1. It doesn't penalize large coefficients; 2. It allows at the same time variable selection, estimation and inference, hence avoiding the need to re-estimate the model after selecting the relevant important predictors and controlling the errors involved in variable selection, in contrast to stepwise methods.

The penalty function in this model is defined by:

$$p'_\lambda(|\beta|) = \lambda \left\{ I(|\beta| \leq \lambda) = \frac{(a\lambda - |\beta|)_+}{(a-1)\lambda} I(|\beta| > \lambda) \right\}$$

The choice of λ is crucial for the performance of the methodology. As λ increases, the number of nonzero components in the vector β decreases. To avoid the danger of overfitting, we have used a tenfold cross validation technique to select λ.

Izzi-Engbeaya C, *et al. BMJ Open Diab Res Care* 2021; 9:e001858. doi: 10.1136/bmjdrc-2020-001858

Supplementary Tables

SUPPLEMENTARY TABLE S1 – Demographic and Clinical Features of 889 Patients Treated for COVID-19 Patients in ICHNT Between March and April 2020 (Univariate Analysis for primary outcome of death or Intensive Care Unit admission within 30 days of swab positive diagnosis).

WHOLE COHORT						
Clinical feature		*Number of patients with data*	*Prevalence (n)*	*Odds ratio of primary outcome (OR)*	*95% confidence interval (CI)*	*p-value*
Female		889	40% (355)	0.69	0.52 to 0.91	**0.01**
Age (years)		889				
	18-49		18% (164)	0.40	0.24 to 0.67	**<0.0001**
	50-59		18% (157)	1	REFERENCE	
	60-69		17% (147)	1.11	0.74 to 1.84	0.55
	70-79		22% (193)	1.33	0.86 to 2.03	0.23
	>80		26% (228)	1.41	0.92 to 2.15	0.21
Weight (kg)		693				
	<60		19% (134)	1.83	1.48 to 2.26	**<0.0001**
	60-90		58% (399)	1	REFERENCE	
	>90		23% (160)	1.02	0.88 to 1.09	0.77
Ethnicity		865				
	White		38% (327)	1	REFERENCE	
	Black		17% (146)	1.21	0.81 to 1.81	0.40
	South Asian		11% (97)	1.04	0.64 to 1.66	>0.9
	All non-White		62% (538)	1.35	1.01 to 1.81	**0.04**
Smoking status		484				
	Current/ex-smoker		189	1	REFERENCE	
	Never smoked		295	1.38	0.93 to 2.03	0.11
Co-morbidities		889		*OR (co-morbidity vs without co-morbidity)*		
	Hypertension		47% (418)	2.41	1.82 to 3.20	**<0.001**
	Diabetes		38% (337)	1.68	1.27 to 2.21	**<0.001**
	Hyperlipidaemia		33% (296)	1.56	1.16 to 2.08	**0.04**
	Ischaemic heart		16% (144)	2.25	1.58 to 3.23	**<0.001**

Izzi-Engbeaya C, *et al. BMJ Open Diab Res Care* 2021; 9:e001858. doi: 10.1136/bmjdrc-2020-001858

	disease					
	Stroke		13% (120)	0.98	0.66 to 1.46	>0.9
	Heart failure		10% (87)	2.49	1.60 to 3.85	**<0.001**
	COPD		9% (80)	1.12	0.71 to 1.81	0.63
	Active cancer		9% (79)	1.15	0.72 to 1.86	0.62
	Pre-COVID-19 eGFR <60mL/kg/min	694		1.40	1.25 to 1.56	**<0.001**
Pre-admission medications		889		*OR (taking specified medication vs not taking the medication)*		
	Statin		42% (373)	1.49	1.13 to 1.97	**0.006**
	Anti-platelet drug		23% (204)		0.92 to 1.75	0.14
	ACE inhibitor		17% (151)	1.57	1.10 to 2.23	**0.013**
	Angiotensin II receptor blocker		13% (116)	1.43	0.95 to 2.12	0.09
CFS Score (1-9)		756				
	1 - 2		40% (300)	1	REFERENCE	
	3 - 4		32% (242)	1.50	1.05 to 2.15	**0.03**
	5 - 6		15% (110)	1.75	1.20 to 2.52	**0.003**
	≥7		13% (104)	2.34	1.46 to 3.75	**0.0004**
eGFR at time of COVID-19 diagnosis (mL/kg/min)		880				
	>90		27% (240)	1	REFERENCE	
	60 - 90		31% (269)	1.60	1.06 to 2.41	**0.02**
	45 - 59		11% (95)	2.35	1.40 to 3.91	**0.001**
	30 - 44		10% (93)	4.28	2.60 to 7.21	**<0.0001**
	15 - 29		9% (78)	3.02	1.78 to 5.16	**<0.0001**
	<15		12% (105)	4.19	2.56 to 6.78	**<0.0001**
Glucose at time of COVID-19 diagnosis		652				
WHOLE COHORT	<10 mmol/L		78% (508)	1	REFERENCE	
	≥10 mmol/L		22% (144)	2.04	1.39 to 2.95	**0.0002**
		394				

PATIENTS WITHOUT DIABETES	<10 mmol/L		94% (369)	1	REFERENCE	
	≥10 mmol/L		6% (25)	3.56	1.60 to 8.62	**0.004***

Supplementary Table S1: 889 patients with confirmed (swab positive) COVID-19 were analysed and univariate odds ratios (OR) against a defined comparator (REFERENCE) are provided for our predefined primary outcome of death or ICU admission within 30 days of diagnosis. ACE inhibitor = angiotensin converting enzyme inhibitor, CBG = capillary blood glucose, CFS = Clinical Frailty Scale, COPD = Chronic obstructive pulmonary disease, DPPIV inhibitor = dipeptidyl peptidase-IV inhibitor, eGFR = estimated glomerular filtration rate, GLP-1RA = glucagon-like peptide-1 receptor agonist, HbA1c = glycated haemoglobin, SGLT2 inhibitor = sodium-glucose co-transporter-2 inhibitor. Significant p-values for odds ratios using Fishers' exact test are shown in **bold.** *Survives correction for age and gender.

SUPPLEMENTARY TABLE S2 – Demographic and Clinical Features of 337 Patients with Diabetes Mellitus treated for COVID-19 in ICHNT Between March and April 2020 (Univariate Analysis for primary outcome of death or Intensive Care Unit admission within 30 days of swab positive diagnosis).

**PATIENTS WITH DIABETES**						
Diabetes diagnosis		889				
	Patients without diabetes		62% (552)	1	REFERENCE	
	Patients with diabetes		38% (337)	1.68	1.27 to 2.12	**0.0003**
Type of diabetes		337				
	Type 2		96% (324)	1	REFERENCE	
	Type 1		4% (13)	0.37	0.11 to 1.27	0.16
Duration of diabetes		189				
	<10 years		47% (88)	1	REFERENCE	
	≥10 years		53% (101)	1.82	1.03 to 3.34	0.054
Diabetes medication at time of COVID-19 diagnosis		337		*OR (taking specified medication vs not taking the medication)*		
	Insulin		31% (108)	0.88	0.55 to 1.42	0.64
	GLP-1RA		1% (5)	0.52	0.09 to 2.60	0.66
	Metformin		49% (169)	1.14	0.74 to 1.76	0.58
	Sulphonylurea		22% (74)	0.85	0.50 to 1.45	0.59
	SGLT2 inhibitor		7% (24)	0.66	0.30 to 1.52	0.40
	DPPIV inhibitor		27% (93)	1.27	0.79 to 2.05	0.39
	Total number of diabetes medications					
	0		18% (57)	1	REFERENCE	
	1		41% (139)	0.91	0.50 to 1.67	0.75
	2		28% (96)	0.97	0.49 to 1.83	>0.9
	≥3		13% (45)	0.95	0.42 to 2.13	>0.9
HbA1c		240				
	<58 mmol/mol		47% (112)	1	REFERENCE	
	≥58 mmol/mol		53% (128)	1.44	0.85 to 2.39	0.19
Glucose at time of		323				

COVID-19 diagnosis						
	<10 mmol/L		56% (181)	1	REFERENCE	
	≥10 mmol/L		44% (142)	1.65	1.07 to 2.56	**0.03**
Average CBG in 1st 72hr after COVID-19 diagnosis		306				
	<10 mmol/L		64% (197)	1	REFERENCE	
	≥10 mmol/L		36% (109)	1.93	1.22 to 3.11	**0.008**
Interaction of Diabetes with IHD		337				
	Without IHD		73% (246)	1	REFERENCE	
	With IHD		27% (91)	1.18	1.03 to 1.37	**0.02**
Interaction of Diabetes with pre-COVID-19 eGFR (mL/kg/min)		337				
	eGFR >80		24% (82)	1	REFERENCE	
	eGFR ≤79		76% (255)	1.53	1.03 to 2.29	**0.04**
Interaction of Diabetes with Hypertension		337				
	Without hypertension		29% (99)	1	REFERENCE	
	With hypertension		71% (238)	1.12	0.80 to 1.58	0.54

Supplementary Table S2: Out of the 889 patients studied in total (see Table S1), this table shows the results for the 337 patients within that group who also had a diagnosis of Diabetes Mellitus (DM). Patients with confirmed (swab positive) COVID-19 were analysed and univariate odds ratios (OR) against a defined comparator (REFERENCE) are provided for our predefined primary outcome of death or ICU admission within 30 days of diagnosis. ACE inhibitor = angiotensin converting enzyme inhibitor, CBG = capillary blood glucose, DPPIV inhibitor = dipeptidyl peptidase-IV inhibitor, eGFR = estimated glomerular filtration rate, IHD = ischaemic heart disease. Significant p-values for odds ratios using Fishers' exact test are shown in **bold.**

SUPPLEMENTARY TABLE S3 - Biochemical Measurements and Clinical Observations for 889 patients admitted with COVID-19 according to whether they met the primary outcome of death or Intensive Care Unit admission within 30 days of swab positive diagnosis.

	**Measurement at time of diagnosis**	**Primary outcome (died/ICU admission within 30 days)**			**Survived/no ICU admission**			**p-value (primary outcome vs survived/no ICU)**
		n	mean	SD	n	mean	SD	
Arterial Blood Gas (ABG) measurement	FiO2 recorded on ABG	260	48.70	26.68	403	29.81	18.2	**<0.0001**
	Lactate (mmol/L)	260	1.83	1.78	403	1.536	1.028	**0.0077**
	pCO2 (kPa)	258	4.93	1.36	402	5.078	2.746	0.4618
	pO2 (kPa)	255	8.81	5.97	397	8.208	5.596	0.1448
	Base excess (mmol/L)	256	-0.41	17.52	397	-0.37	4.953	0.1049
	pH	259	7.41	0.097	401	7.421	0.08202	0.222
	Bicarbonate (mmol/L)	256	23.28	5.69	400	24.05	4.415	0.0962
Laboratory results	WCC ($x10^9$/L)	320	8.61	4.09	561	7.95	4.635	**0.0015**
	Hb (g/L)	320	125.70	23.15	562	129.3	20.96	**0.0264**
	Platelet count ($x10^9$/L)	319	218.20	104.70	562	238.4	105.5	**0.0024**
	Neutrophils ($x10^9$/L)	318	7.42	7.22	562	6.346	8.549	**<0.0001**
	Lymphocytes ($x10^9$/L)	317	0.98	0.997	562	1.219	1.206	**<0.0001**
	D-dimers (ng/ml)	221	3895	5292	332	2445	3671	**<0.0001**
	Ferritin (ng/mL)	233	2122	5214	358	1313	1824	**<0.0001**
	Na (mmol/L)	320	138.30	6.231	560	137	7.424	**0.043**
	K (mmol/L)	309	4.33	0.75	534	4.213	0.5569	0.0783
	eGFR (mL/min)	319	51.14	29.67	560	65.66	27.47	**<0.0001**
	eGFR pre-COVID19 baseline (mL/min)	236	60.38	29.93	457	72.64	46.48	**<0.0001**
	ALT (U/L)	295	53.08	155.10	515	39.28	57.06	0.6581
	CRP (mg/L)	314	156.9	104.40	542	94.33	88.34	**<0.0001**
	CK (U/L)	206	641.9	1184	311	391.8	1179	**<0.0001**
	LDH (U/L)	186	602.5	626	291	398.8	230.2	**<0.0001**
	BNP (pg/mL)	184	423.1	1451	267	192.2	762.7	**<0.0001**
	Procalcitonin (ng/mL)	33	20.5	69.04	49	0.538	1.527	**<0.0001**
	HbA1c - most recent (mmol/mol)	160	54.18	20.28	274	53.27	21.22	0.4003
Glucose	CBG – at time of COVID19 diagnosis	269	10.04	5.54	416	8.312	5.398	**<0.0001**
	Average CBG in first 72hr of COVID-19 diagnosis	244	9.256	3.59	332	7.795	3.187	**<0.0001**
Clinical observations at	Temperature (°C)	311	37.38	1.19	558	37.26	1.016	**0.0441**

diagnosis	Respiratory Rate (Breaths per minute)	312	25.54	8.82	556	22.03	6.049	**<0.0001**
	Heart Rate (beats per minute)	314	95.91	20.34	558	92.42	18.37	**0.0093**
	Systolic BP (mmHg)	316	134.8	27.49	560	131.2	23.74	**0.0452**
	Diastolic BP (mmHg)	316	73.53	16.06	559	75.67	13.81	**0.0321**
	NEWS at arrival	300	6	3.44	540	4.05	3.642	**<0.0001**

Supplementary Table S3: Unadjusted comparisons are shown for patients who met the primary outcome (death or ICU admission within 30 days of diagnosis) against those who survived and did not have an ICU admission: n = number of patients for whom that data set is available. Note the total number of patients arriving at the primary outcome was 323. ABG = arterial blood gas, ALT = alanine aminotransferase, BNP = brain natriuretic peptide, BP = blood pressure, CBG = capillary blood glucose, CRP = C-reactive protein, CK = creatinine kinase, eGFR = estimated glomerular filtration rate, FiO2 = fraction of inspired oxygen, Hb = haemoglobin, HbA1c = glycated haemoglobin, K = potassium, LDH = lactate dehydrogenase, Na = sodium, NEWS = National Early Warning Score, WCC = white cell count.

SUPPLEMENTARY TABLE S4 - Biochemical Measurements and Clinical Observations for 889 patients admitted with COVID-19 according to whether or not they had a pre-existing diagnosis of Diabetes Mellitus

	**Measurement at time of diagnosis**	**Patients without diabetes mellitus (no DM)**			**Patients with diabetes mellitus (DM)**			**p-value (no DM vs DM)**
		n	mean	SD	n	mean	SD	
Arterial Blood Gas (ABG) measurement	FiO2 recorded on ABG	403	36.4	23.2	260	38.51	24.64	0.2867
	Lactate (mmol/L)	402	1.58	1.4	261	1.765	1.347	**0.0031**
	pCO2 (kPa)	401	4.96	1.28	259	5.117	3.321	0.8276
	pO2 (kPa)	397	8.27	5.45	255	8.71	6.183	0.5349
	Base excess (mmol/L)	397	0.0348	5.05	256	-2.048	6.781	**0.0003**
	pH	401	7.43	0.0813	259	7.401	0.09645	**0.001**
	Bicarbonate (mmol/L)	398	24.3	4.69	258	22.83	5.233	**0.0022**
Laboratory results	WCC ($x10^9$/L)	545	8.12	4.61	337	8.308	4.194	0.276
	Hb (g/L)	546	130	22.1	337	124.8	21.09	**0.0006**
	Platelet count ($x10^9$/L)	545	232	111	337	230.4	95.88	0.6622
	Neutrophils ($x10^9$/L)	545	6.28	4.18	336	7.472	11.98	0.0805
	Lymphocytes ($x10^9$/L)	545	1.17	1.34	335	1.079	0.7064	0.8785
	D-dimers (ng/ml)	336	2556	3714	218	3742	5305	**0.0096**
	Ferritin (ng/mL)	358	1553	2368	234	1751	4896	0.6944
	Na (mmol/L)	544	138	7.62	337	136.9	5.944	**0.0008**
	K (mmol/L)	524	4.18	0.603	320	4.378	0.6716	**<0.0001**
	eGFR (mL/min)	544	67	26.4	336	49.64	30.14	**<0.0001**
	eGFR pre-COVID19 baseline (mL/min)	411	75.4	47.2	283	58.38	30.34	**<0.0001**
	ALT (U/L)	494	46.7	113	317	40.54	88.75	0.0657
	CRP (mg/L)	527	106	94	330	135.8	104.3	**<0.0001**
	CK (U/L)	307	510	1320	211	464.5	960.5	0.6773
	LDH (U/L)	293	480	413	185	475.4	482.1	0.9935
	BNP (pg/mL)	279	201	754	173	422.4	1489	0.0688
	Procalcitonin (ng/mL)	55	8.96	49.4	27	7.774	33.11	**0.0469**
	HbA1c - most recent (mmol/mol)	194	39.8	7.14	240	64.73	21.66	**<0.0001**
Glucose	CBG – at time of COVID19 diagnosis	375	6.97	2.07	311	11.43	7.145	**<0.0001**
	Average CBG in first 72hr of COVID-19 diagnosis	273	6.9	1.77	303	9.776	3.969	**<0.0001**
Clinical observations at diagnosis	Temperature (°C)	537	37.3	1.07	333	37.27	1.101	0.7922
	Respiratory Rate (Breaths per	539	23.3	7.41	330	23.34	7.278	0.6356

	minute)							
	Heart Rate (beats per minute)	541	93	19.1	332	94.72	19.24	0.256
	Systolic BP (mmHg)	543	130	24.5	334	136.1	25.99	**0.0011**
	Diastolic BP (mmHg)	542	75.9	14.5	334	73.24	14.84	**0.0053**
	NEWS at arrival	523	4.63	3.95	321	4.938	3.234	0.0515

Supplementary Table S4: Unadjusted comparisons are shown for patients who had diabetes mellitus (n=337) and those who did not (n=552). n = number of patients for whom that data set is available. Note the total number of patients arriving at the primary outcome was 323. ABG = arterial blood gas, ALT = alanine aminotransferase, BNP = brain natriuretic peptide, BP = blood pressure, CBG = capillary blood glucose, CRP = C-reactive protein, CK = creatinine kinase, eGFR = estimated glomerular filtration rate, FiO2 = fraction of inspired oxygen, Hb = haemoglobin, HbA1c = glycated haemoglobin, K = potassium, LDH = lactate dehydrogenase, Na = sodium, NEWS = National Early Warning Score, WCC = white cell count.

SUPPLEMENTARY TABLE S5 - Biochemical Measurements and Clinical Observations for 337 patients who had Diabetes Mellitus and were admitted with COVID-19, according to whether they met the primary outcome of death or Intensive Care Unit admission within 30 days of swab positive diagnosis.

	**Measurement at time of diagnosis**	**Patients with Diabetes who suffered the primary outcome (died/ICU admission within 30 days) (n=149)**			**Patients with Diabetes who survived/no ICU admission (n=188)**			**p-value (primary outcome vs survived/no ICU)**
		n	mean	SD	n	mean	SD	
Arterial Blood Gas (ABG) measurement	FiO2 recorded on ABG	120	48.03	26.32	140	30.36	19.83	**<0.0001**
	Lactate (mmol/L)	120	1.868	1.608	141	1.678	1.075	0.5536
	pCO2 (kPa)	119	4.787	1.252	140	5.397	4.355	0.1177
	pO2 (kPa)	117	10.04	7.493	138	7.583	4.533	**0.003**
	Base excess (mmol/L)	118	-2.86	7.946	138	-1.354	5.533	0.1905
	pH	119	7.399	0.09554	140	7.403	0.09751	0.7566
	Bicarbonate (mmol/L)	119	22.14	5.748	139	23.42	4.689	0.0853
Laboratory results	WCC ($x10^9$/L)	149	8.411	3.807	188	8.226	4.485	0.2624
	Hb (g/L)	149	123.4	22.92	188	126	19.51	0.2594
	Platelet count ($x10^9$/L)	149	211.4	84.43	188	245.4	101.8	**0.0011**
	Neutrophils ($x10^9$/L)	148	7.732	9.643	188	7.267	13.56	**0.008**
	Lymphocytes ($x10^9$/L)	147	0.9442	0.5931	188	1.184	0.7689	**0.0006**
	D-dimers (ng/ml)	108	4703	6244	110	2800	3995	**0.0298**
	Ferritin (ng/mL)	113	2230	6798	121	1304	1727	0.107
	Na (mmol/L)	149	137.7	6.015	188	136.3	5.834	**0.0346**
	K (mmol/L)	145	4.404	0.7085	175	4.357	0.6406	0.563
	eGFR (mL/min)	148	43.41	29.4	188	54.55	29.89	**0.0008**
	ALT (U/L)	142	50.71	127.6	175	32.29	30.81	0.1423
	CRP (mg/L)	147	162.9	102	183	114.1	101.3	**<0.0001**
	CK (U/L)	102	716.6	1304	109	228.6	296.3	**0.0002**
	LDH (U/L)	87	584	658.6	98	379	190.7	**0.0036**
	BNP (pg/mL)	94	570.6	1944	80	248.4	592.5	**0.0483**
	Procalcitonin (ng/mL)	16	12.68	42.85	11	0.64	1.096	**0.0299**
	HbA1c - most recent (mmol/mol)	99	63.56	20.14	141	65.55	22.71	0.968
Glucose	CBG – at time of COVID19 diagnosis	140	12.28	6.537	171	10.74	7.555	**0.0021**
	Average CBG in first 72hr of COVID-19 diagnosis	141	10.5	3.937	162	9.141	3.898	**0.0004**
Clinical observations at diagnosis	Temperature (°C)	146	37.33	1.201	187	37.22	1.018	0.3678
	Respiratory Rate (Breaths per minute)	146	25.34	8.627	184	21.76	5.53	**<0.0001**

	Heart Rate (beats per minute)	146	97.2	21.48	186	92.78	17.1	**0.0376**
	Systolic BP (mmHg)	147	137.3	27.68	187	135.1	24.62	0.4514
	Diastolic BP (mmHg)	147	72.5	15.78	187	73.82	14.07	0.4181
	NEWS at arrival	142	6.07	3.37	183	4.06	2.837	**<0.0001**

Supplementary Table S5: Unadjusted comparisons are shown for patients with diabetes (total n=337) who met the primary outcome (death or ICU admission within 30 days of diagnosis, n=149) against those who survived and did not have an ICU admission (n=188): n = number of patients for whom that data set is available. Note the total number of patients arriving at the primary outcome was 323. ABG = arterial blood gas, ALT = alanine aminotransferase, BNP = brain natriuretic peptide, BP = blood pressure, CBG = capillary blood glucose, CRP = C-reactive protein, CK = creatinine kinase, eGFR = estimated glomerular filtration rate, FiO2 = fraction of inspired oxygen, Hb = haemoglobin, HbA1c = glycated haemoglobin, K = potassium, LDH = lactate dehydrogenase, Na = sodium, NEWS = National Early Warning Score, WCC = white cell count.

BMJ Publishing Group Limited (BMJ) disclaims all liability and responsibility arising from any reliance placed on this supplemental material which has been supplied by the author(s)

SUPPLEMENTARY TABLE S6 - Multivariate Regression Analysis with SCAD sensitivity analysis to determine the important contributing regressors against the Primary Outcome of Death/ICU Admission within 30 Days of COVID-19 Diagnosis (n=719 patients)

Regressor	*Estimate*	*SE*	*p-value*	*Marginal Effect (%)*
Age	0.031	0.012	**0.009**	**0.5**
Gender	0.595	0.249	**0.017**	**10.3**
Ischaemic Heart Disease	0.002	0.017	0.893	0.04
Hypertension	0.147	0.331	0.657	2.6
Antiplatelet drug	-0.383	0.354	0.278	-6.7
Clinical Frailty Score	0.147	0.072	**0.043**	**2.5**
White cell count	0.035	0.049	0.472	0.6
Haemoglobin	-0.009	0.014	0.528	-0.2
Platelets	-0.004	0.002	**0.038**	**-0.1**
Neutrophils	0.000	0.015	0.977	<0.001
Serum sodium	0.044	0.025	0.077	0.8
Serum potassium	0.048	0.167	0.772	0.8
eGFR on diagnosis	-0.008	0.011	0.460	-0.1
C-reactive protein	0.002	0.002	0.298	0.0
Temperature at diagnosis	-0.052	0.276	0.851	-0.9
Respiratory rate on diagnosis	0.033	0.023	0.149	0.6
Heart Rate on diagnosis	0.006	0.008	0.427	0.1
Systolic Blood Pressure	0.011	0.006	**0.041**	**0.2**
Diastolic Blood Pressure	-0.004	0.010	0.657	-0.1
NEWS at diagnosis	-0.004	0.080	0.959	-0.1
Inspired Oxygen delivered at diagnosis	-0.013	0.007	0.087	-0.2
Oxygen saturations at diagnosis	-0.035	0.030	0.236	-0.6
Maximum inspired oxygen required	0.067	0.005	**<0.0001**	**1.2**

Supplementary Table S6**:** Unselected multivariate logistic (Logit) analysis of all variables that were collected for patients admitted with swab positive COVID 19 with the primary outcome of death or ICU admission within 30 days (719 patients) – that data is shown in Table 1. Thereafter a sensitivity analysis was performed to select the truly important variables in our regression. We used a Smoothly Clipped Absolute Deviation (SCAD) regularised regression technique which delivered 23 variables independently selected to be the most important drivers of the model, and new p-values are recreated to adjust for this analysis. Refer to Table 1 for details on the other statistical measures presented. eGFR = estimated glomerular filtration rate, NEWS = National Early Warning Score.

Izzi-Engbeaya C, *et al. BMJ Open Diab Res Care* 2021; 9:e001858. doi: 10.1136/bmjdrc-2020-001858

SUPPLEMENTARY TABLE S7 - Multivariate Regression Analysis with SCAD sensitivity analysis to determine the important contributing regressors against the Primary Outcome of Death/ICU Admission within 30 Days of COVID-19 Diagnosis in Patients with Diabetes Mellitus (n=268 patients)

Regressor	*Estimate*	*SE*	*p-value*	*Marginal Effect (%)*
Age	0.041	0.017	**0.016**	**0.9**
Gender	0.024	0.105	0.822	0.5
White cell count	-0.001	0.027	0.970	0.0
Haemoglobin	-0.012	0.011	0.265	-0.3
Platelets	-0.007	0.003	**0.008**	**-0.2**
Neutrophils	0.000	0.006	0.971	0.0
Serum sodium	0.000	0.008	0.982	0.0
eGFR on diagnosis	-0.008	0.007	0.236	-0.2
C-reactive protein	0.000	0.002	0.836	0.0
Capillary blood glucose on diagnosis	0.024	0.030	0.422	0.6
Temperature on diagnosis	-0.017	0.077	0.824	-0.4
Respiratory rate on diagnosis	0.040	0.031	0.196	0.9
Heart rate on diagnosis	-0.004	0.010	0.682	-0.1
Systolic blood pressure	0.004	0.011	0.713	0.1
Diastolic blood pressure	0.006	0.022	0.800	0.1
Inspired oxygen delivered at diagnosis	0.003	0.012	0.773	0.1
Oxygen saturations at diagnosis	-0.005	0.047	0.908	-0.1
Maximum inspired oxygen required	0.057	0.008	**<0.0001**	**1.3**

Supplementary Table S7: Unselected multivariate logistic (Logit) analysis of all variables that were collected for patients admitted with swab positive COVID 19 who had diabetes mellitus, as applied to the primary outcome of death or ICU admission within 30 days (268 patients) – that data is shown in Table 2. Thereafter a sensitivity analysis was performed to select the truly important variables in our regression. We used a Smoothly Clipped Absolute Deviation (SCAD) regularised regression technique which delivered 19 variables independently selected to be the most important drivers of the model, and new p-values are recreated to adjust for this analysis. Refer to Table 1 for details on the other statistical measures presented. eGFR = estimated glomerular filtration rate.

Supplementary References

1. Tibshirani, R., *Regression Shrinkage and Selection via the Lasso.* Journal of the Royal Statistical Society. Series B (Methodological), 1996. **58**(1): p. 267-288.
2. Tibshirani, R., *The lasso method for variable selection in the Cox model.* Stat Med, 1997. **16**(4): p. 385-95.
3. Yengo, L., et al., *Impact of statistical models on the prediction of type 2 diabetes using non-targeted metabolomics profiling.* Mol Metab, 2016. **5**(10): p. 918-925.
4. Hastie, T., R. Tibshirani, and R. Tibshirani, *Best Subset, Forward Stepwise, or Lasso? Analysis and Recommendations Based on Extensive Comparisons.* Preprint at https://arxiv.org/abs/1707.08692, 2020.
5. Fan, J. and R. Li, *Variable selection via nonconcave penalized likelihood and its oracle properties.* Journal of the American Statistical Association, 2001. **96**(456): p. 1348-1360.
